# Structural insights into the activation of the divisome complex FtsWIQLB

**DOI:** 10.1038/s41421-023-00629-w

**Published:** 2024-01-03

**Authors:** Lili Yang, Yujiao Chen, Shenghai Chang, Chongrong Shen, Xin Wang, Changbin Zhang, Zhibo Zhang, Bi-Sen Ding, Zhaoming Su, Haohao Dong, Xiaodi Tang

**Affiliations:** 1 Department of Laboratory Medicine, State Key Laboratory of Biotherapy, National Clinical Research Center for Geriatrics, West China Hospital, Sichuan University, Chengdu, Sichuan China; 2 Center of Cryo Electron Microscopy, Zhejiang University, Hangzhou, Zhejiang China

**Keywords:** Electron microscopy, Cell division

Dear Editor,

Cell division is a fundamental process for the growth and reproduction of organisms. Most bacteria proliferate through binary fission, which occurs by the ingrowth of the cell membrane and the peptidoglycan (PG) cell wall to form a septum at midcell^1^. The synthesis of the septal PG is catalyzed by a multi-protein complex named divisome, containing the core components PG glycosyltransferase (GT) FtsW and PG transpeptidase (TP) FtsI that are responsible for septal glycan chain polymerization and crosslinking, respectively^2,3^ (Fig. 1a). The action of the septal PG synthase FtsWI is strictly regulated by divisome regulatory proteins, including FtsN, FtsA, FtsEX, and FtsQLB^4–7^. Previous mutagenic studies suggest that FtsN initiates a signaling cascade for FtsWI activation through FtsQLB^8,9^. However, the mechanism by which FtsWI is activated is not fully understood. Here, we determined a cryo-EM structure of FtsWI bound to the regulatory proteins FtsQLB at 3.3 Å and performed mutagenic studies to reveal the molecular details for understanding the mechanism of divisome activation.

**Fig. 1:**
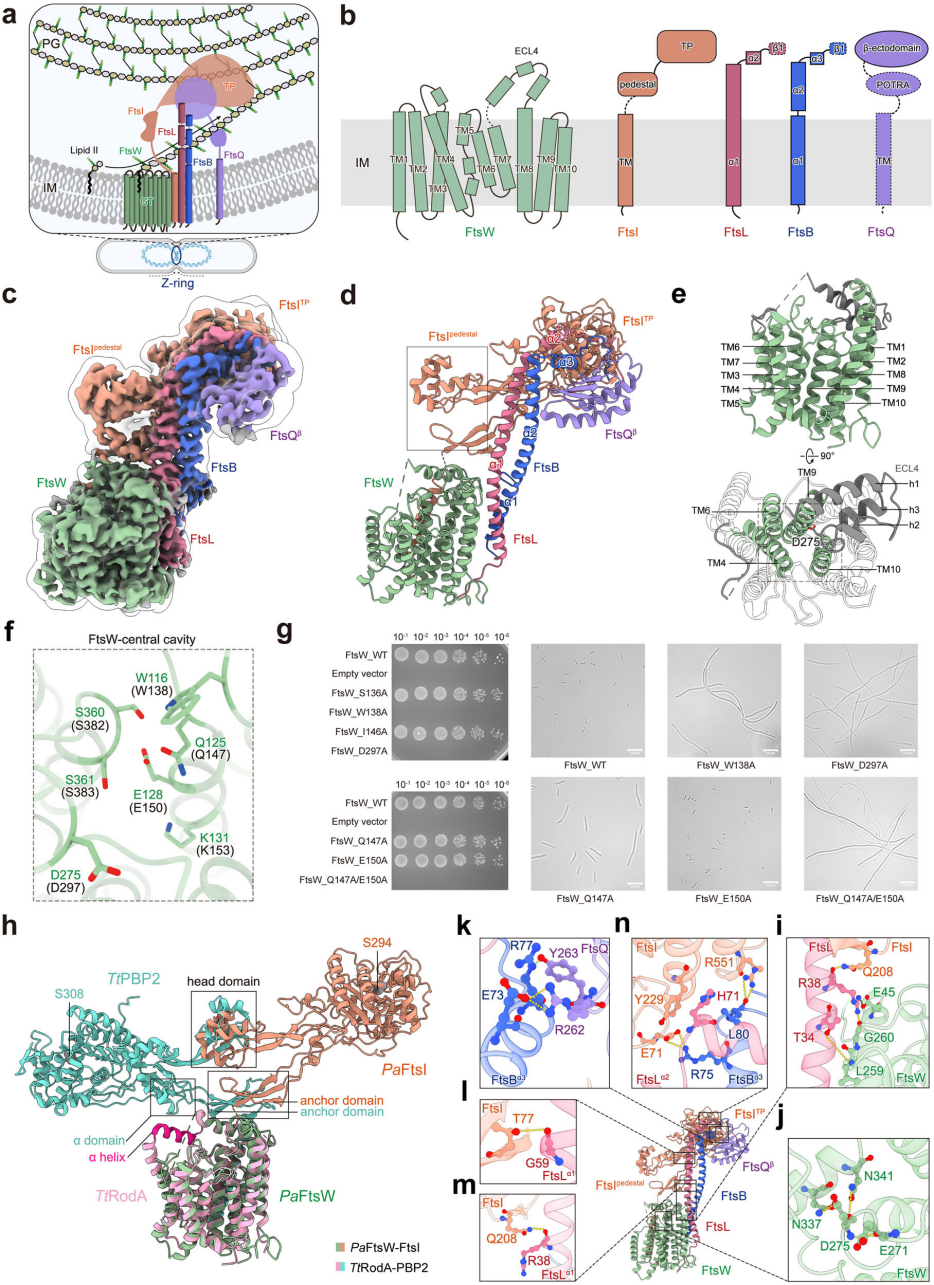
Structural characterization of the *Pa*FtsWIQLB complex. **a** Schematic diagram of peptidoglycan synthesis by the divisome complex FtsWIQLB. **b** Topology of FtsW, FtsI, FtsL, FtsB, and FtsQ. **c**, **d** Cryo-EM map (**c**) and structure (**d**) of the *Pa*FtsWIQLB complex. Flexible loops are shown as dashed lines. **e** Transmembrane helices of FtsW from the side and top views, showing ECL4 (grey). **f** The conserved residues present in the central cavity of FstW (corresponding residues in *E. coli* are in parentheses). **g** Cell viability and microscopic phenotypes of FtsW variants with mutated cavity residues. **h** Structural superimposition of *Pa*FtsW-FtsI and *Tt*RodA-PBP2 (PDB:6PL5) with overlapped FtsW and RodA as reference. **i** The interactions between FtsW and FtsL. **j** The putative catalytic residue D275 of FtsW interacts with residues within the central cavity. **k**–**n** Interactions between components of the FtsWIQLB complex are highlighted in boxes. Hydrogen bonds and salt bridges are indicated with yellow or black dashed lines.

The FtsWIQLB complex from *Pseudomonas aeruginosa* was expressed, purified, and visualized by a cryo-electron microscope (cryo-EM) (Supplementary Table S1). The high-resolution cryo-EM map allowed unambiguous assignments for the FtsWIQLB complex, except for FtsQ, of which only the C-terminal β ectodomain (FtsQ^β^) was resolved (Fig. 1b–d; Supplementary Fig. S1). FtsB and FtsL form a long α-helical coiled-coil at the center of the structure, with its N-terminus associated with the transmembrane (TM) α-helical bundle of FtsW, and its C-terminus extending into the periplasmic space, where it interacts with FtsQ and FtsI through their ectodomains. FtsI contains an N-terminal single TM segment (FtsI^TM^) that interacts with FtsW in the TM region and a ‘trophy-like’ C-terminal structure composed of a transpeptidase (TP) domain and a pedestal domain (FtsI^TP-pedestal^), interacting with the FtsBL coiled coil in the periplasm (Fig. 1c, d).

FtsW belongs to the shape, elongation, division, and sporulation (SEDS) family, which functions in cognate pairs with type B penicillin-binding protein (bPBP) family transpeptidases for PG synthesis (Supplementary Fig. S2). FtsW comprises ten TM α-helices (TM1–TM10) and a large extracellular loop (Fig. 1b, e). The overall configuration of FtsW is highly homologous to the reported structure of another SEDS protein, RodA (PDB: 6BAR), which functions with PBP2 in PG synthesis during cell elongation^10^ (Supplementary Fig. S3a). The structure of FtsW is arranged with TM4, TM6, TM9, and TM10 at the center, forming a quadruple helical core, while the remaining TMs are situated around the periphery (Fig. 1e). TM9 inserts into the central pocket with an angle, together with TM4, TM6, and TM10 forming an outward-open cavity (Fig. 1e). The cavity of FtsW contains highly conserved polar residues, with their polar side chains exposed in the lumen (Fig. 1f). Variants of FtsW carrying single or double alanine substitutions of the conserved residues exhibited growth defects and filamentous phenotypes (Fig. 1g; Supplementary Fig. S3b–d). These results implicate their importance in the function of FtsW, probably in the binding of the amphipathic substrate, Lipid II, during glycan chain polymerization. In our structure, the largest extracellular loop 4 (ECL4) of FtsW, located between TM7 and TM8, is partially resolved. The segment near TM7 (residues 225–236) is disordered, and the resolved portion contains three consecutive α-helices (h1–h3) that are horizontally stacked on top of TM1–2 and 8–10, covering half of the central cavity of FtsW (Fig. 1e). The loop between h2 and h3 (h2 loop) in ECL4 contains the putative catalytic residue D275 that positions right above the opening of the central cavity (Fig. 1e, f). Previous studies have found that the mutant FtsW^D275A^ (*Ec*FtsW^D297A^) showed a dominant-negative phenotype and abolishment of the catalytic activity of FtsW in both *E*. *coli* and *P. aeruginosa*^3,11^. This result is consistent with the deficient phenotype observed in our *Ec*FtsW^D297A^ mutant (Fig. 1g; Supplementary Fig. S3b–d). FtsW interacts with FtsI mainly through the TM helix^12^. Deletion of FtsI^TM^ failed to pull down the co-expressed FtsW (Supplementary Fig. S4a–c), indicating that the transmembrane interface is essential for their complexation. The pedestal domain of FtsI (FtsI^pedestal^) is positioned on the top of ECL4 of FtsW, showing a second interface in the periplasmic space, however, no specific interaction is detected here (Supplementary Fig. S4a).

Structural comparison with the homologous structure of RodA–PBP2 (PDB: 6PL5)^13^ reveals a marked reorientation of FtsI in the FtsWIQLB structure (Fig. 1h). Specifically, when aligning FtsW with RodA, FtsI rotates about 107° along the membrane plane relative to the position of PBP2, resulting in opposite orientations of the TP domain of the bPBP proteins (Supplementary Fig. S4d). In the RodA–PBP2 assembly, PBP2 lies nearly horizontally on top of RodA, with its TP domain positioned close to the membrane. In contrast, FtsI^TP^ is positioned significantly towards the periplasm, engaging with the ectodomain of FtsLB (Fig. 1d, h). This elevated repositioning of FtsI^TP^ away from the cytoplasmic membrane suggests a conformation that may be conducive to PG layer synthesis. Such a spatial arrangement would potentially allow PG chain polymerization and crosslinking to occur simultaneously at the membrane interface and the outer PG layer without steric hindrance (Fig. 1a, h). Furthermore, the interface between the PBP2^pedestal^ and the ECL4 of RodA is larger than that between FtsW and FtsI. The α domain and the anchor domain of PBP2^pedestal^ make multiple contacts with RodA’s ECL4, which stabilize the otherwise disordered segment of ECL4 in the FtsW structure (residues 215–224) into an α-helix (Supplementary Fig. S4e, f). This stabilized α-helix in the RodA structure bridges with an adjacent α-helix, covering the entire cavity of RodA^13^ (Supplementary Fig. S4f). Conversely, the corresponding segment in ECL4 of RodA appears disordered compared to the organized h2 helix of FtsW (Fig. 1e; Supplementary Fig. S4f). In our structure, the h2 loop of FtsW is stabilized by FtsL (Fig. 1i). It has been reported that this loop around h2 at the periplasmic interface is important for the activity of SEDS–bPBP complex^9^, implicating that the h2 loop stabilization observed in our structure may be crucial for FtsW–FtsI activation. Moreover, this stabilized h2 loop also positions the putative catalytic residue D275 to interact with conserved residues in the central cavity of FtsW (Fig. 1j). In contrast, the corresponding catalytic residue D255 in the RodA structure is orientated outwards, precluding interactions (Supplementary Fig. S4f).

FtsB and FtsL form the coiled-coil primarily through hydrophobic interactions (Supplementary Fig. S5a, b). FtsQ interacts with FtsBL through the C-terminal α3 helix of FtsB (FtsB^α3^) (Fig. 1d, k). Deletion of the α3 domain or the C-terminus of FtsB abolished complex formation with FtsQ (Supplementary Fig. S4c). FtsI is associated from the side of FtsL, with FtsI^pedestal^ interacting with the long α1 helix of FtsL (FtsL^α1^) and FtsI^TP^ interacting with FtsL^α2^ and FtsB^α3^ (Fig. 1l–n). In the TM region, FtsLB associates with FtsW through only one hydrogen bond (Supplementary Fig. S5c), whereas at the membrane-proximal region FtsL makes multiple interactions with the h2 loop of FtsW, including a salt bridge and a hydrogen bond between FtsL^R38^ and FtsW^E45^ and FtsW^G260^, respectively (Fig. 1i). The FtsL^R38D^ mutant has been reported to cause FtsWI activation defect in vitro^7^, emphasizing its role in stabilizing the h2 loop for FtsWI activation.

It is proposed that FtsQLB assumes an angled orientation within the membrane. This postulation arises from the observation that the TM segments of FtsLB and FtsQ are at different levels in the crystal structure of isolated FtsQLB (PDB: 8HHG), implying a tilt to reconcile both TM segments within the same membrane plane^14^. Relative to the position of the ectodomains of the complexes, the FtsLB coil in our structure has stretched to a relatively perpendicular conformation to the membrane plane when compared with the crystal structure of FtsQLB (Supplementary Fig. S5d). We speculate that this perpendicular configuration of FtsLB could maximize the distance of the transpeptidase (interacted via the FtsLB^ectodomain^) of the synthase complex from the membrane. Although FtsQ is not fully resolved in our structure, the consistency in the conformation of the FtsQ ectodomain suggests a maintained spatial configuration, anchoring the protein in a triangular formation with FtsLB (Supplementary Fig. S5d). The geometrical positioning of FtsQLB is presumed to provide stable support, promoting the erect posture of the entire complex.

To summarize, structural comparisons of FtsWIQLB with RodA–PBP2 and isolated FtsQLB provide implications to the mechanism of FtsWI activation by FtsQLB. The binding of the regulatory complex FtsQLB induces the allosteric changes in both FtsW and FtsI to facilitate their activation, including stabilizing the ECL4 loop of FtsW and positioning FtsI^TP^ to the site of PG synthesis. While composing this manuscript, a parallel study reported a cryo-EM structure of FtsWIQLB (PDB: 8BH1) at 3.7Å^15^, showing a high resemblance to our structure (Supplementary Fig. S5e). Interestingly, using AlphaFold2, they predicted a new conformational state of the complex in which FtsI^TP^ rotated an additional 30° to a higher position, matching the distance of the PG layer from the bacterial membrane. Given that the current configuration of the FtsLB helical coil is at nearly maximal stretch (perpendicular to the membrane plane), further rotation of FtsI may be induced by the conformational changes within the ectodomains, which requires further exploration. The septal PG synthesis is a key step in cell division. As PG synthesis is the primary antibiotic target, Fts complex holds significant antimicrobial potential. Our study sheds light on the activation mechanism of the SEDS–bPBP complex and provides a structural basis for future structure-based drug design.

### Supplementary information

Supplementary information, Figures and Table

## Data Availability

The cryo-EM map is deposited in the Electron Microscopy Data Bank (accession code: EMD-17356). The model is deposited in the Protein Data Bank (accession code: 8P1U).
