# Supplementary material for: Structural insights into the activation of the divisome complex FtsWIQLB
Source: Cell Discov. 2024 Jan 3;10:2. doi: 10.1038/s41421-023-00629-w (PMC10764723; doi:10.1038/s41421-023-00629-w)
Supplement: Supplementary file 1 — Supplementary information, Figures and Table [file 41421_2023_629_MOESM1_ESM.pdf]

Supplementary Information for

Structural insights into the activation of the divisome complex FtsWIQLB

Lili Yang[1,4], Yujiao Chen[1,4], Shenghai Chang[2,3,4], Chongrong Shen[1], Xin Wang[1], Changbin zhang[1], Zhibo Zhang[1], Bi-Sen Ding[1], Zhaoming Su[1 *], Haohao Dong[1*] & Xiaodi Tang[1*]

[1]Department of Laboratory Medicine, State Key Laboratory of Biotherapy, National Clinical Research Center for Geriatrics, West China Hospital, Sichuan University, Chengdu, China;

[2]Department of Biophysics and Department of Pathology of Sir Run Run Shaw Hospital, Zhejiang University, School of Medicine, Hangzhou, Zhejiang 310058, China;

[3]Center of Cryo Electron Microscopy, Zhejiang University, Hangzhou, China;

[4]These authors contributed equally to this work.

[*]Correspondence: tangxiaodi@scu.edu.cn (X.T.); haohaodong@scu.edu.cn (H.D.); zsu@scu.edu.cn (Z.S.).

This file includes:

Materials and Methods

Data availability

Additional references

Supplementary Figs. S1 to S5

Supplementary Table S1

Materials and methods

No statistical methods were used to predetermine the sample size. The experiments were not randomized. The investigators were not blinded to allocation during carry out experiment and outcomes assessment.

Plasmid construction

For obtaining the protein complex, the genes encoding the *ftsWI* complex were cloned from the *Pseudomonas aeruginosa* genome DNA by polymerase chain reaction (PCR) and ligated into the modified pCOLADuet-1 vector. A small ubiquitin-like modifier (SUMO) protein was fused at the N-terminus of FtsW, and a Twin-Strep-Tag (TST)[1] attached to the C-terminus of FtsI. The codon optimized *ftsQLB* genes were also amplified by PCR and ligated into the pETDuet-1vector, which a SUMO protein was fused at the N-terminus of FtsQ. For the functional assays, the *ftsW* gene from *Escherichia coli MG1655* were amplified and ligated into the pTrc99a vector, with the N-terminus fused with a SUMO protein and the C-terminus tagged with a TST. All constructs were confirmed by sequencing.

Protein expression and purification

The recombinant plasmids of pCOLADuet-1-SUMO-FtsWI-TST and pETDuet-1-SUMO-FtsQLB were co-transformed into the *E. coli* C43 (DE3) cells (Novagen). Single colonies were inoculated at 37°C in LB (Luria-Bertani) medium supplemented with appropriate antibiotics (50 μg ml^{-1} kanamycin, 100 μg ml^{-1} Ampicillin). When the optical density of the culture reached 1.0 at a wavelength of 600 nm ($OD_{600\ nm}$), overexpression of the complex was induced by adding 200 mM Isopropyl β-D-

Thiogalactoside (IPTG) at 20°C overnight. Cell pellets were collected by centrifugation at 4,000 rpm and resuspended in lysis buffer A (50 mM HEPES, pH 7.0, 150 mM NaCl and 20 mM $MgCl_2$). Cells were lysed and ultracentrifuged at 35,000 rpm for 1 h to separate the membrane fractions. The membrane fractions were resuspended in buffer B (20 mM HEPES, pH 7.0, 500 mM NaCl and 20% Glycerol) containing 0.5% (w/v) n-Dodecyl-β-D-maltoside (DDM, Anatrace) and 0.5% (w/v) Lauryl maltose neopentyl glycol (LMNG, Anatrace) at room temperature for 30 min. The suspension was ultracentrifuged again at 35,000 rpm for 25 min to remove the insolubilized membrane fraction. The supernatant was loaded onto a Streptavidin Agarose (MedChemExpress) affinity column which was pre-equilibrated with 10 ml buffer B containing 0.02% (w/v) LMNG. Binding was allowed for 1h at room temperature with gentle agitation. The column was washed with 10 column volume (CV) buffer B containing 0.02%(w/v) LMNG. The protein complex was eluted with buffer B containing 50 mM D-Biotin and 0.01% (w/v) LMNG. The eluted sample was concentrated and applied to a size exclusion chromatography column (Superdex 200 Increase 10/300 GL column, GE Healthcare) in buffer C (20 mM HEPES, pH 7.5, 300 mM NaCl) containing 0.01% (w/v) LMNG. The peak fractions were collected and concentrated to 4-10 mg ml^{-1} for cryo-EM sample preparation.

Complex stabilization assays

For the FtsI(ΔTM), FtsB(ΔC-terminus) and FtsB(Δα3) mutants, the pCOLADuet-1-FtsWI and pETDuet-1-FtsQBL vector were used as a template to introduce mutations or truncations. FtsW was tagged with His-tag, FtsI and FtsL were tagged with Myc-tag,

and FtsQ and FtsB were tagged with Flag-tag for detecting the expression and complex formation by western blot. All mutants were created using site-directed mutagenesis and confirmed by sequencing. The TST tagged FtsI was purified by Streptavidin Agarose affinity column to pull down all other protein components of the complex, following the method as described above. Purified mutants were quantified using the Bicinchoninic Acid Assay (BCA) to equalize the amount of the total protein of each sample. The presence of each protein was detected by western blot using mouse anti-His (1:3000 dilution; SAB2702218, Sigma), anti-Myc (1:1000 dilution; A5963, Sigma) and anti-Flag monoclonal antibody (1:5000 dilution; F3165, Sigma). Protein bands pulled down with the purification of FtsI were visualized by chemiluminescence in a photo imager (Bio-Rad).

***ftsW* knock-out strain construction**

The *E. coli* MG1655 chromosomal genome was modified by inserting a rescue copy of *ftsW* sequence under *araC-araBAD control* into the *attB* site in front of the *ybhc* gene using the CRISPR-Cas9 genomic editing method to generate a derivate strain[2]. The original chromosomal *ftsW* gene was depleted by λ-Red recombination[3] and replaced by an antibiotic resistant gene. Positive colonies were confirmed by PCR and gene sequencing.

Cell viability assays and Microscopy

The pTrc99a-SUMO-*Ec*FtsW construct were used as a template to introduce mutations. The C terminus of *Ec*FtsW was inserted with a TST for detecting protein expression by western blot. The single or double *Ec*FtsW mutants were transformed into the *E. coli*

ftsW depletion strain and grown on LB plates supplemented with 100 μg ml^{-1} ampicillin, 25 μg ml^{-1} chloramphenicol and 0.02% arabinose for 12 hours. Single transformed colonies were picked and cultured in fresh LB liquid medium supplemented with antibiotics and 0.02% arabinose overnight. Cultures were collected by centrifugation and washed three times with fresh LB to remove arabinose, then resuspended with 1ml fresh LB. For cell viability assays, cells were diluted with fresh LB to an $OD600_{nm}$ = 0.5 and incubated on LB agar plates with further tenfold serial dilution at 37°C for 15 hours.

The phenotype of the *Ec*FtsW mutants were observed under a phase contrast microscope (Olympus BX ML51-N), the washed cells were diluted 1:100 and transferred into fresh LB with antibiotics. After 5 hours at 30°C, bacteria were transferred onto clean glass slide and fixed by alcohol lamp for photographing. Protein expression of the *Ec*FtsW mutants transformed in *E. coli* C43(DE3) competent cells were purified and detected by western blot.

Cryo-grid preparation and EM data collection

The purified FtsWIQLB complex (3 μl) at a concentration of ~4 mg $ml^{-1}$was applied to Quantifoil holey carbon grids onto glow-discharged (R1.2/1.3, 300 mesh, copper). Grids were blotted for 3.5 s with the environmental chamber set at 95% humidity and flash frozen in liquid ethane using a Vitrobot Mark IV (Thermo Fisher). The grids were imaged in a 300 keV Titan Krios electron microscope (Thermo Fisher) and equipped with a Selectris energy filter with a slit width of 10 eV and a Falcon4 camera (Thermo Fisher). The software SerialEM[4] was used to record the cryo-EM movies in super-

resolution with 0.93 Å/ pixel. All data were acquired at a nominal magnification of ×130,000, and the defocus ranging from−1.0 to −2.0 μm. Each image was exposed for 8 s and dose fractionated into 40 frames.[5]

Image processing and 3D reconstructions

Beam-induced motion correction of image stacks was carried out using MotionCor2[6] Software to generate 2× binned average micrographs and dose-weighted micrographs with a pixel size of 0.93 Å. The contrast transfer function parameters of these average micrographs were estimated by Gctf.[7] All following image processing steps were performed with cryoSPARC[8] or RELION.[9] For the FtsWIQLB structures, 7,354,539 particles were automatically selected, and two-dimensional and three-dimensional classifications were carried out to select consistent particle classes. Finally, 340,758 particles were selected for three-dimensional refinement. A reported resolution map at 3.3 Å was generated after post-processing with a B factor of −114.5 $Å^2$.

Model building and refinement

The cryo-EM map of FtsWIQLB was built using the predicted structures by Alphafold of FtsW, FtsI, FtsQ, FtsL, FtsB as the starting model to dock into the 3.3 Å resolution map in Coot[10]. The high-resolution cryo-EM maps for most of the structures allowed side-chain assignments, except for the weak densities in FtsQ POTRA-TM and some loops and turns in FtsW and FtsI.

The entire structure of FtsWIQLB was further manually adjusted and automatically refined using PHENIX[11] with phenix.real_space_refines. The statistics of the

refinements are listed in Supplementary Table S1. Structural figures were prepared by Chimera[12] and Chimera X.

References

1 Schmidt, T. G. & Skerra, A. The Strep-tag system for one-step purification and high-affinity detection or capturing of proteins. *Nat Protoc* **2**, 1528-1535, doi:10.1038/nprot.2007.209 (2007).

2 Ruiz, N., Gronenberg, L. S., Kahne, D. & Silhavy, T. J. Identification of two inner-membrane proteins required for the transport of lipopolysaccharide to the outer membrane of Escherichia coli. *Proc Natl Acad Sci U S A* **105**, 5537-5542, doi:10.1073/pnas.0801196105 (2008).

3 Baba, T. *et al.* Construction of Escherichia coli K-12 in-frame, single-gene knockout mutants: the Keio collection. *Mol Syst Biol* **2**, 2006 0008, doi:10.1038/msb4100050 (2006).

4 Mastronarde, D. N. SerialEM: A Program for Automated Tilt Series Acquisition on Tecnai Microscopes Using Prediction of Specimen Position. *Microscopy and Microanalysis* **9**, 1182-1183, doi:10.1017/s1431927603445911 (2003).

5 Obtaining cryo-EM structures by scanning transmission electron microscopy. *Nat Methods* **19**, 1179-1180, doi:10.1038/s41592-022-01587-z (2022).

6 Zheng, S. Q. *et al.* MotionCor2: anisotropic correction of beam-induced motion for improved cryo-electron microscopy. *Nat Methods* **14**, 331-332, doi:10.1038/nmeth.4193 (2017).

7 Zhang, K. Gctf: Real-time CTF determination and correction. *J Struct Biol* **193**, 1-12, doi:10.1016/j.jsb.2015.11.003 (2016).

8 Punjani, A., Rubinstein, J. L., Fleet, D. J. & Brubaker, M. A. cryoSPARC: algorithms for rapid unsupervised cryo-EM structure determination. *Nature Methods* **14**, 290-296, doi:10.1038/nmeth.4169 (2017).

9 Scheres, S. H. Semi-automated selection of cryo-EM particles in RELION-1.3. *J Struct Biol* **189**, 114-122, doi:10.1016/j.jsb.2014.11.010 (2015).

10 Emsley, P., Lohkamp, B., Scott, W. G. & Cowtan, K. Features and development of Coot. *Acta Crystallogr D Biol Crystallogr* **66**, 486-501, doi:10.1107/S0907444910007493 (2010).

11 Adams, P. D. *et al.* PHENIX: a comprehensive Python-based system for macromolecular structure solution. *Acta Crystallogr D Biol Crystallogr* **66**, 213-221, doi:10.1107/S0907444909052925 (2010).

12 Pettersen, E. F. *et al.* UCSF Chimera--a visualization system for exploratory research and analysis. *J Comput Chem* **25**, 1605-1612, doi:10.1002/jcc.20084 (2004).

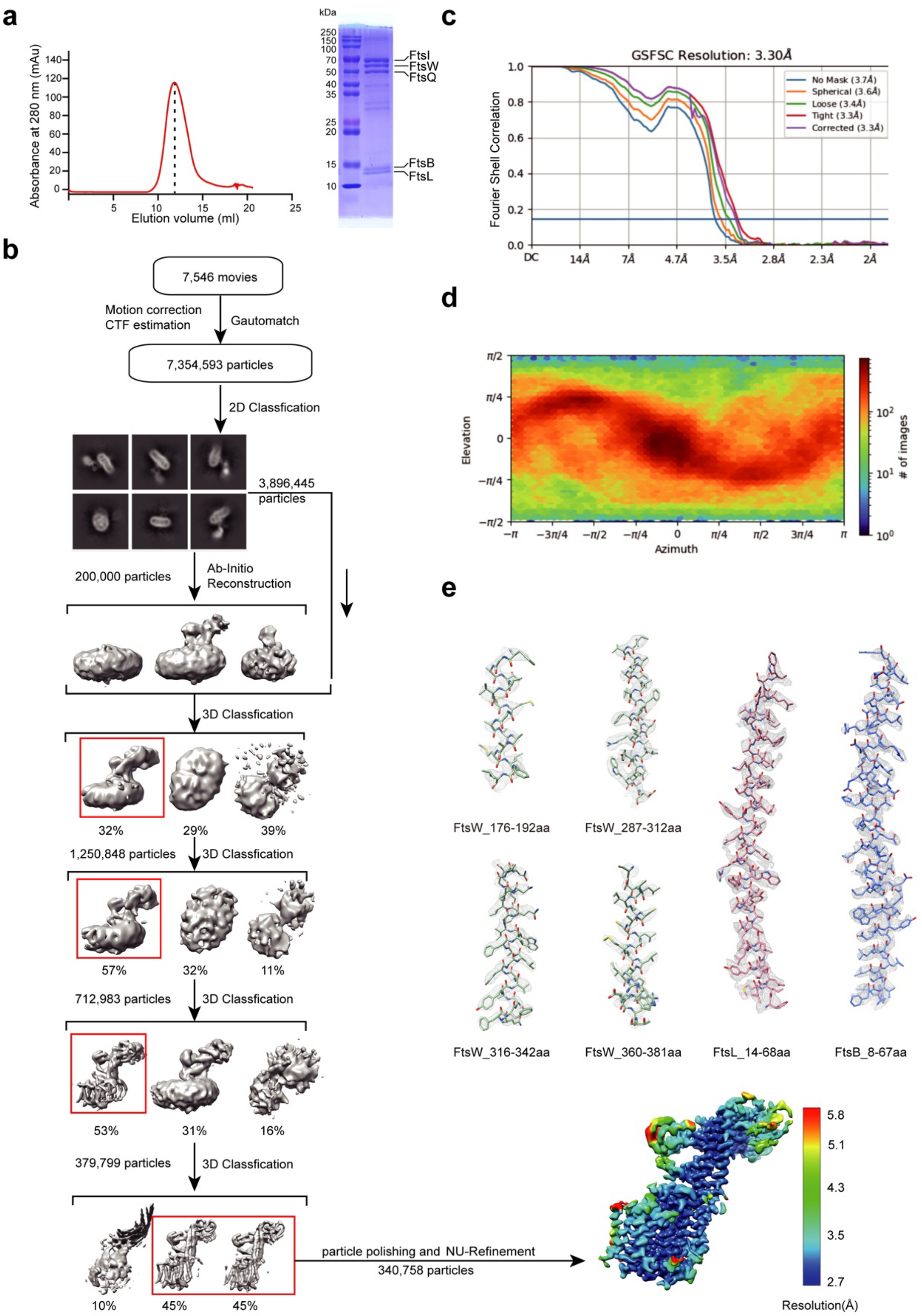

Supplementary Fig. S1. Flow charts for cryo-EM structure determination of FtsWIQLB complex. a Size-exclusion chromatography and Coomassie brilliant blue staining of purified *Pa*FtsWIQLB. **b** Scheme of data collection, two- and three- dimensional classifications, refinements for FtsWIQLB. **c** Gold-standard FSC curves of the final EM maps. **d** Angular distribution heatmap at the bottom panel calculated in cryoSPARC. **e** Cryo-EM maps with the atom model for partial transmembrane helices of FtsW, FtsB and FtsL.

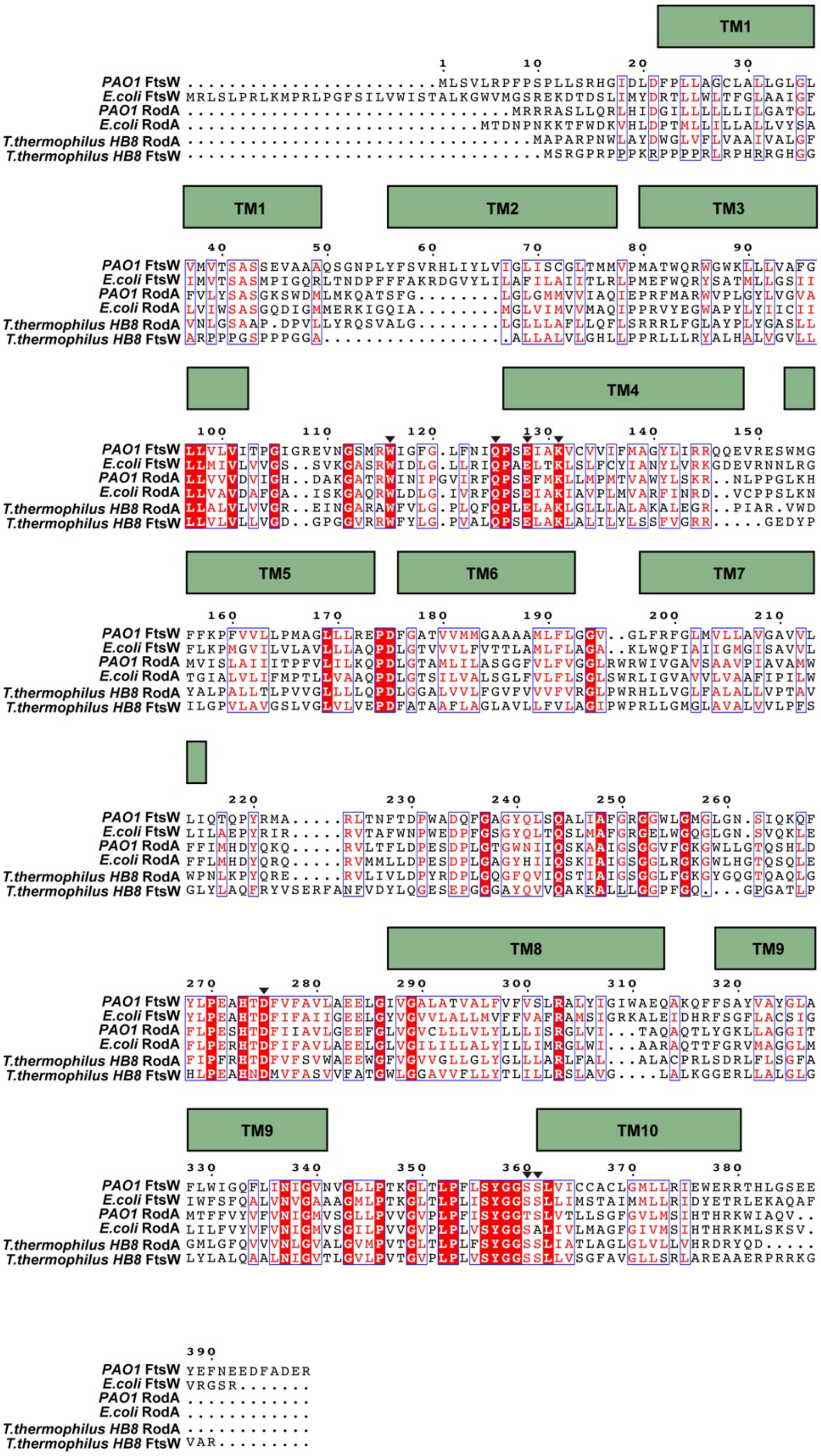

Supplementary Fig. S2. FtsW and RodA sequence alignment from different species. The result of the alignment of FtsW and RodA sequences from *P.aeruginosa*, *E. coli K12* and *T.thermophilus HB8* using Clustal Omega, with representative examples displayed. Highly conserved residues across all 399 residues are shown in red. The ones that selected for mutagenesis in functional assays were indicated by black arrows. Based on the structure, the secondary structure elements of *P.aeruginosa* FtsW are indicated on the top (green boxes).

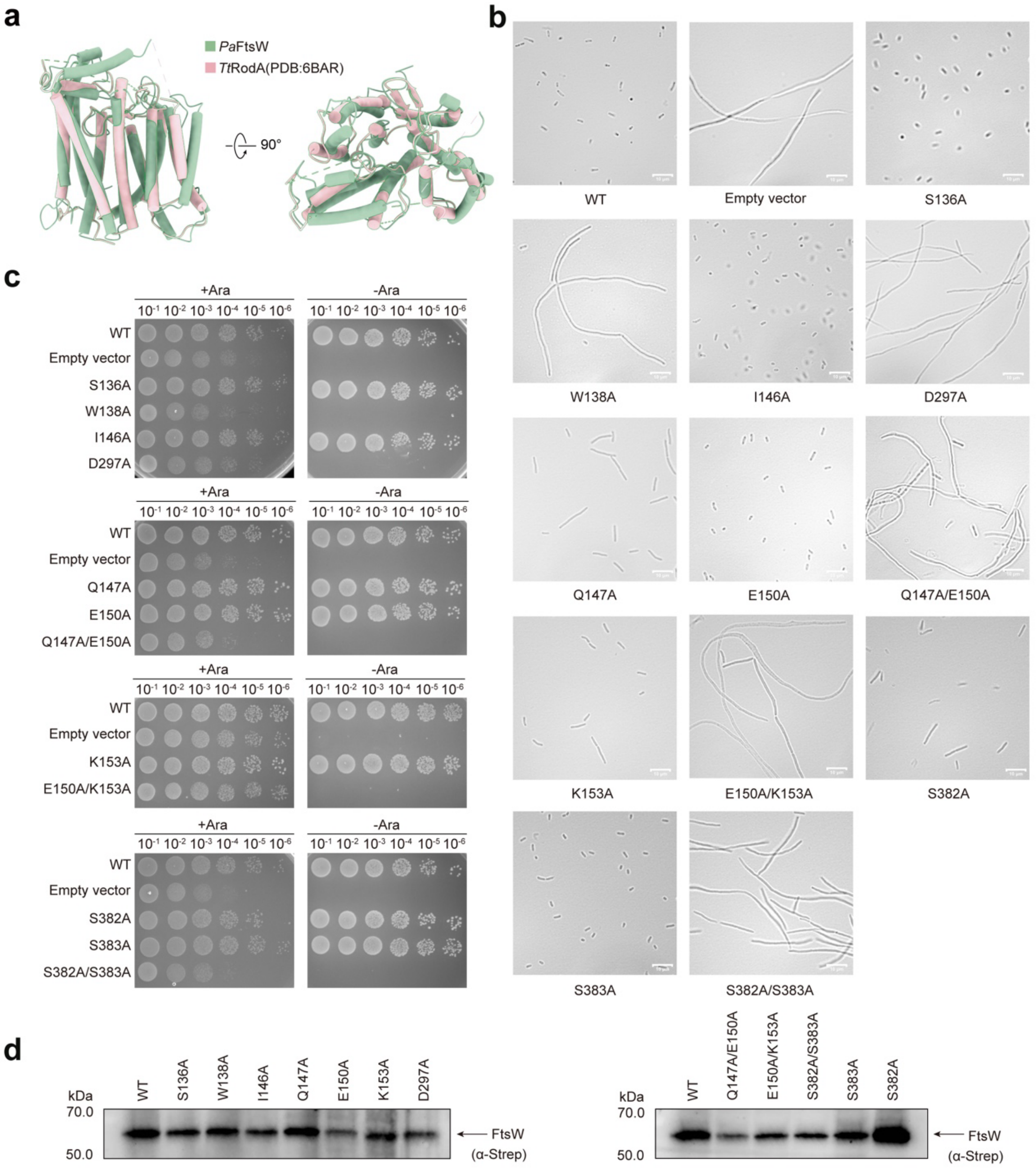

Supplementary Fig. S3. Identification of functional residues in the cavity of FtsW. a Structural comparison of FtsW (*P.aeruginosa*) with RodA (*T.thermophilus HB8*) (PDB:6BAR). **b** Micrographs showing the phenotypes of wildtype or FtsW mutants. Scale bar: 10 μm. **c** Cell viability assays of wildtype or FtsW mutants grown on agar plates in the presence or absence of arabinose. **d** Western blot detection of FtsW mutant proteins. Data in **c-d** are representative results from n=3 independent experiments.

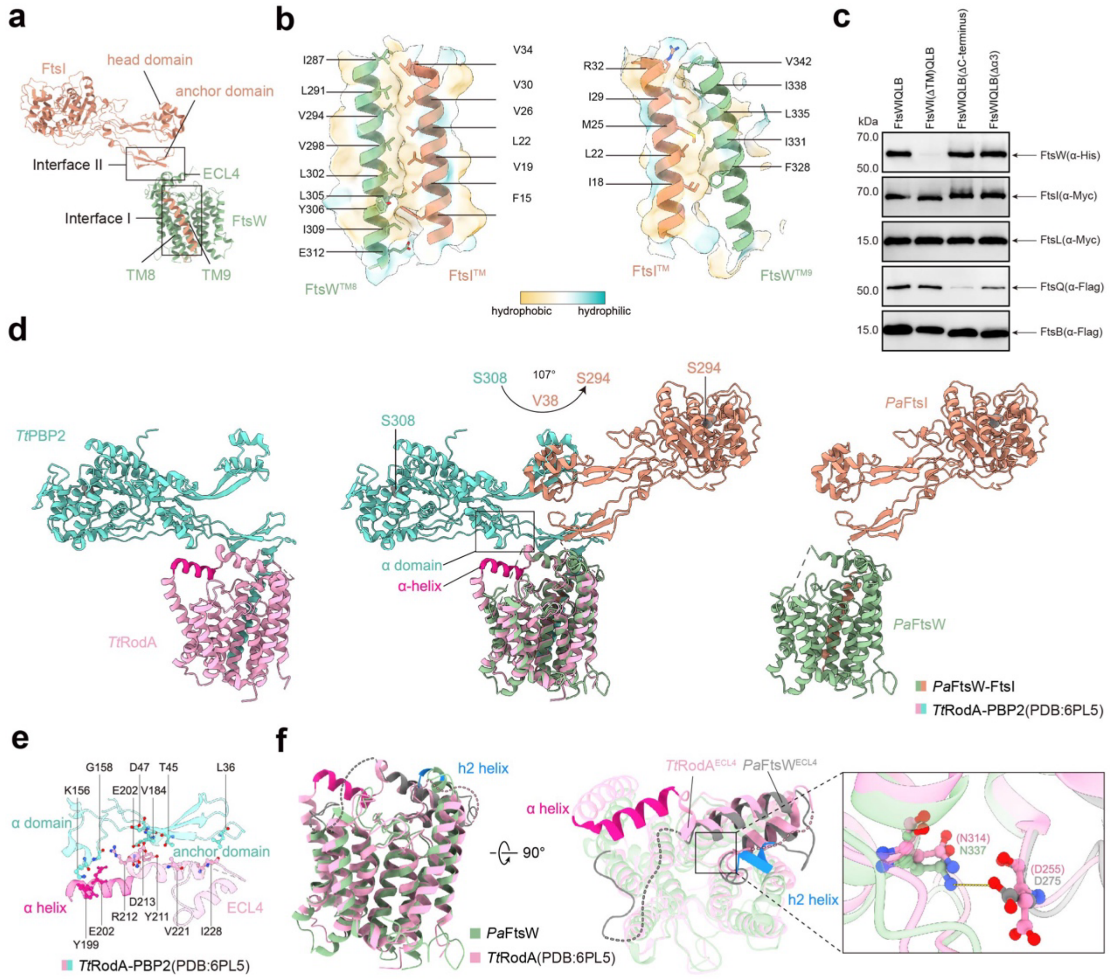

Supplementary Fig. S4. Structural characterization of FtsW-FtsI and the homologous *Tt*RodA-PBP2 structure. **a** FtsWI complex showing two distinct interfaces between FtsW and FtsI. **b** Transmembrane segment of FtsI is shown as ribbon and transparent hydrophobic surfaces to highlight its hydrophobic interactions with the TM8 (left) or TM9 (right) of FtsW. **c** Western blot of the pulled down proteins by purified FtsI with or without mutations in FtsI or FtsB. FtsWI(ΔTM)QLB: deletion the TM of FtsI; FtsWIQLB(ΔC-terminus): deletion aa67-aa97 of FtsB; FtsWIQLB(Δα3): deletion aa70-aa78 of FtsB. **d** Structural superimposition of *Pa*FtsW-FtsI and *Tt*RodA-PBP2 complex structure (PDB:6PL5). **e** Residues involved in protein contact at the interface between RodA ECL4 and PBP2^{pedestal} from the structure of *Tt*RodA-PBP2 (PDB:6PL5) are highlighted. **f** Superimposition of *Pa*FtsW and *Tt*RodA from the complex structure (PDB:6PL5), showing structural resemblance with a root mean square deviation (rmsd) of 1.15 Å over 217 aligned Cα atoms but distinct ECL4 configurations. Zoomed central cavity showing distinct orientations of the putative catalytic residues D275 and D255 of FtsW and RodA, respectively. D275 in the cavity of FtsW interacts with the conserved residue N337.

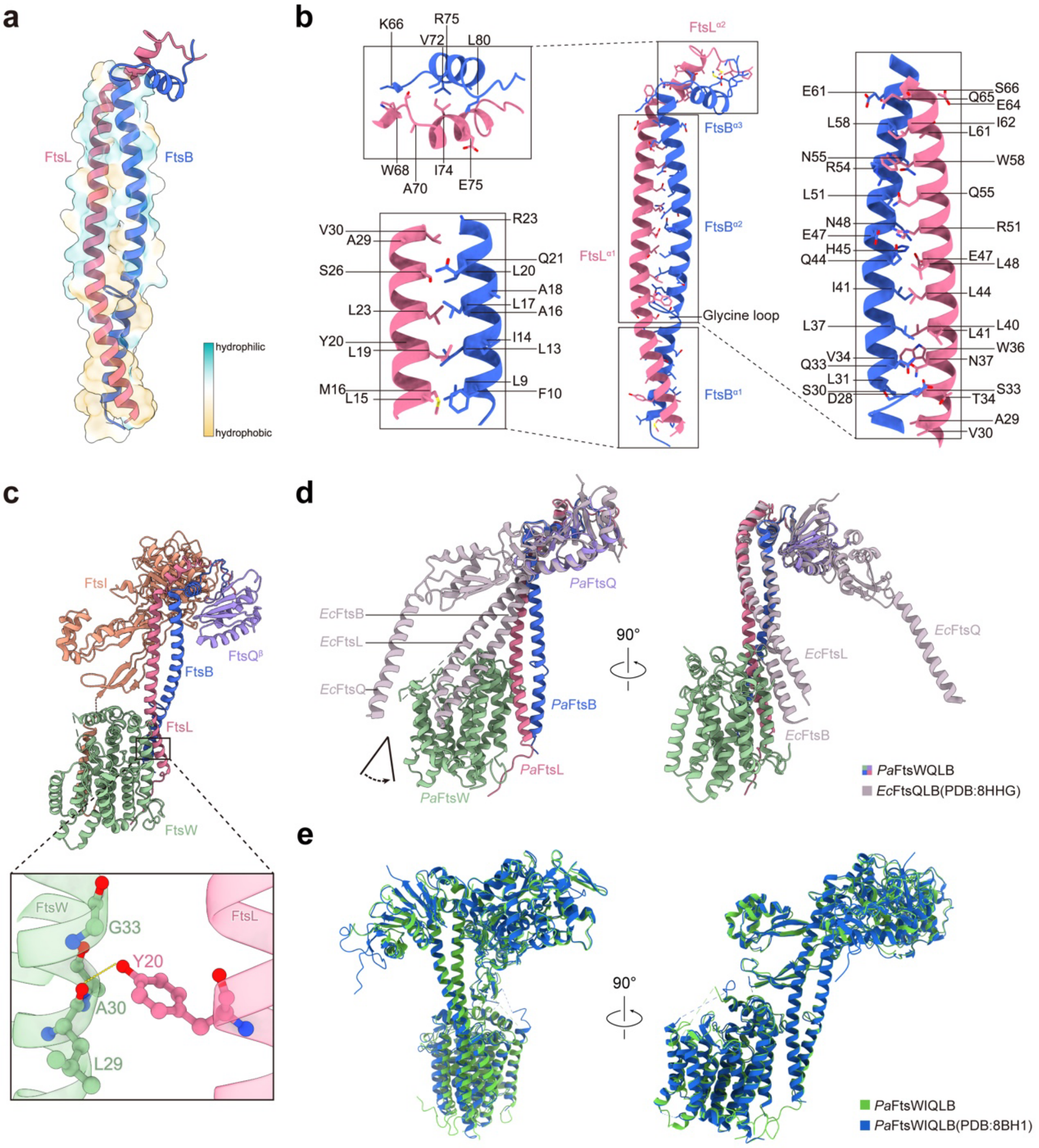

Supplementary Fig. S5. Structural characterization of the regulatory complex FtsQLB. **a, b** the surface of the FtsLB structure showing hydrophobic interface (orange) (a). The interacting residues between FtsL and FtsB are shown as stick (b). **c** Y20 of FtsL interacts with the TM1 of FtsW, Hydrogen bond is indicated by yellow dashed line. **d** Structural superposition of our *Pa*FtsWQLB structure and the crystal structure of *Ec*FtsQLB (PDB:8HHG), exhibiting the conformational changes in FtsLB. **e** Structural superposition of our *Pa*FtsWQLB structure and the reported cryo-EM structure of FtsWIQLB (PDB:8BH1), showing structural resemblance with rmsd of 0.78 Å over 508 aligned Cα atoms.

Supplementary Table S1. Cryo-EM data collection, refinement, and validation statistics.

	FtsWIQLB (EMD-17356, PDB 8P1U)
**Data collection and processing**	
Magnification	130000
Voltage (kV)	300
Detector	Falcon4
Electron exposure (e^-/Å^2)	50
Defocus range (μm)	-1.0 to -2.0
Pixel size (Å)	0.93
Symmetry imposed	*C1*
Initial particle images (no.)	7,354,593
Final particle images (no.)	340,758
Map resolution (Å)	3.3
FSC threshold	0.143
Map resolution range (Å)	2.5-6.3
**Refinement**	
Initial model used	alphafold
Map sharpening *B* factor (Å^2)	-114.5
Model composition	
Non-hydrogen atoms	9149
Protein residues	1179
Ligands	0
B factors (Å^2)	
Protein	53.45
Ligand	0
R.m.s. deviations	
Bond lengths (Å)	0.003
Bond angles (°)	0.558
**Validation**	
MolProbity score	1.81
Clashscore	10.2
Poor rotamers (%)	0.21
Ramachandran plot	
Favored (%)	95.87
Allowed (%)	4.13
Disallowed (%)	0
